# Sucralose Exposure During Pregnancy Elevates Gestational Diabetes Risk via Gut Microbiota‐Metabolic Axis in Mice

**DOI:** 10.1155/jdr/8638903

**Published:** 2026-05-13

**Authors:** Jiajia Song, Juhui He, Zhaoxia Liang

**Affiliations:** ^1^ Department of Obstetrics, Women′s Hospital, School of Medicine, Zhejiang University, Hangzhou, Zhejiang, China, zju.edu.cn; ^2^ Zhejiang Key Laboratory of Maternal and Infant Health, Hangzhou, Zhejiang, China

**Keywords:** gestational diabetes mellitus, gut microbiota, metabolism, sucralose

## Abstract

Sucralose, a widely used nonnutritive artificial sweetener, has gained increasing popularity during pregnancy due to its low‐calorie properties. The influence of sucralose on gestational diabetes mellitus (GDM) risk via alterations in gut microbiota composition is not yet well understood. This study sought to explore how gestational sucralose exposure influences GDM development in mice through gut microbiota dysbiosis, impaired intestinal barrier, and metabolic disorders. As an exploratory extension, we also preliminarily assessed early growth in offspring. In our experimental design, pregnant mice were administered sucralose solution to evaluate GDM incidence. Fecal samples were collected for 16S rRNA sequencing and untargeted metabolomic analysis. To establish causality, gut microbiota from sucralose‐exposed pregnant mice was transplanted into control pregnant mice to assess subsequent GDM development and alterations in fecal 16S rRNA profiles. Additionally, we monitored postpartum glucose metabolism in sucralose‐treated pregnant mice and tracked offspring body weight changes. Our results demonstrated that sucralose exposure significantly increased GDM incidence, accompanied by higher glucose levels and diminished insulin sensitivity. Furthermore, sucralose administration caused significant gut microbiota imbalance. It reduced beneficial taxa like *Prevotellaceae UCG-001* and *Lachnospiraceae UCG-001* while increasing the proinflammatory taxon *Parasutterella*. These changes strongly correlated with key fecal metabolites, including 5‐aminopentanoic acid. Notably, maternal sucralose consumption during pregnancy also affected offspring body weight. These findings collectively indicate that gestational sucralose exposure elevates GDM risk in mice through the gut microbiota‐metabolism axis, providing critical scientific evidence regarding the safety of low‐calorie sweeteners in clinical applications during pregnancy.

## 1. Introduction

Gestational diabetes mellitus (GDM) signifies an important metabolic issue during pregnancy, with a rapidly increasing global prevalence [1, 2], and certain high‐risk cohorts exhibiting prevalence rates exceeding 30% [3, 4]. It not only threatens maternal health but also elevates risks of adverse fetal outcomes, including macrosomia, preterm delivery, and birth trauma [5, 6]. Additionally, advancing age substantially elevates the chances of Type 2 diabetes and heart disease [7–9]. The long‐term consequences for offspring include heightened susceptibility to obesity and metabolic syndrome [10–12]. Thus, early identification and intervention of GDM are vital for promoting maternal and child health.

The association between dietary factors and GDM has recently garnered considerable scientific attention. NNS, commonly consumed by pregnant women for weight and glycemic control, have emerged as a focus of investigation [13]. Cross‐national studies report that 18%–45% of pregnant women consume NNS [13]. Additionally, given the increasing prevalence of NNS‐containing food products, up to 40% of pregnant women may inadvertently ingest NNS despite no intentional [14]. In certain populations, nearly 98% of expectant mothers regularly consume NNS‐sweetened beverages, with sucralose being the most prevalent, accounting for 96% of total intake [15–17]. Limited evidence suggest that sucralose may increase GDM risk and disturb glucose and lipid metabolism [18–20], but the underlying mechanism has not been elucidated. This paucity of systematic research underscores critical knowledge gaps in understanding the pathogenesis of sucralose‐mediated GDM, necessitating urgent investigation.

Research has shown that dietary habits significantly affect the composition and functionality of gut microbiota. Earlier research has thoroughly examined how different dietary elements affect gut microbiota [21, 22]. Sucralose has been reported to alter gut microbial structure and metabolism, thereby affecting host metabolic homeostasis and increasing susceptibility to diabetes and cardiovascular diseases [23, 24]. Furthermore, experimental evidence suggests that sucralose may exert metabolic effects through modulation of short‐chain fatty acid production [25].

Concurrently, a growing body of research has established a strong relationship between gut microbiota imbalance and GDM development [26, 27]. Specific alterations in microbial taxa have been linked to GDM development during early pregnancy [28]. GDM patients typically show lower gut microbial *α*‐diversity, along with fewer beneficial bacteria and more pathogenic taxa, which aggravate insulin resistance and glucose intolerance [29, 30]. Building upon this theoretical framework, we hypothesize that sucralose causes an imbalance in gut microbiota, leading to weakened intestinal barrier function and low‐level inflammation, and disturbs systemic metabolism, ultimately promoting GDM development.

To explore the mechanism linking gestational sucralose exposure, gut microbiota, and GDM, this study utilized a mouse model combined with 16S rRNA gene sequencing, untargeted metabolomics, and FMT. To our knowledge, this is the first comprehensive study demonstrating that sucralose induces GDM by triggering microbiota dysbiosis and subsequent intestinal and metabolic disorders. These findings support scientific guidance for dietary advice and microbiota‐targeted prevention strategies for GDM during pregnancy.

## 2. Materials and Methods

### 2.1. Experimental Animals

Female C57BL/6 J mice, aged 11 weeks, were sourced from the Zhejiang Provincial Laboratory Animal Center and maintained under controlled environmental conditions with unrestricted access to food and water. After acclimating for 1 week, the mice were exposed to experimental procedures. All animal experiments adhered to protocols approved by the Laboratory Animal Welfare & Ethics Committee of Women′s Hospital, School of Medicine, Zhejiang University (Approval No. AE 20250090).

### 2.2. Experimental Design and Group Distribution

This study utilized two complementary experimental models to elucidate the mediating role of gut microbiota in sucralose‐induced GDM:

1. Conventional pregnant mouse model (direct sucralose exposure): Pregnant mice were divided randomly into three different groups: Control (CON), low‐dose sucralose (SUC 0.24), and high‐dose sucralose (SUC 0.72). Each group consisted of 14 mice.
2. FMT model: Germ‐free mice were transplanted with fecal microbiota from the three donor groups described above and assigned to three parallel groups: FMT‐CON, FMT‐SUC 0.24, and FMT‐SUC 0.72. Each group consisted of 10 mice.

Detailed procedures for FMT are described in Section 2.5.

### 2.3. Drug Preparation

Female C57BL/6 J mice were mated with male counterparts in a 1:1 ratio. Daily vaginal plug inspections were performed at 09:00 from the second day, with the presence of a plug marking embryonic day 0.5 (E0.5). The days of gestation were denoted by E, so E12.5 and E18.5 represent the 12.5th and 18.5th days of embryonic development, respectively. Pregnant mice were randomly assigned into three experimental groups: control, low‐dose sucralose (SUC 0.24), and high‐dose sucralose (SUC 0.72). Sucralose concentrations were calculated using human safety thresholds set by the EFSA (15 mg/kg body weight) and the US FDA (5 mg/kg body weight) [31, 32], adjusted for murine metabolism via the HED. Sucralose was administered ad libitum in drinking water at concentrations of 24 mg/L (defined as low‐dose) or 72 mg/L (defined as high‐dose) from E0.5 throughout gestation. The dosage per mouse was estimated by weekly monitoring of water consumption per cage and average body weight during the second trimester of pregnancy, enabling calculation of the approximate daily intake in mg/kg body weight/day, as detailed in Table S1.

### 2.4. OGTT and ITT

To assess gestational glucose metabolism, an OGTT was conducted at E12.5 after a 12‐h fasting period, with water available freely. Following the measurement of baseline body weight and fasting blood glucose levels, mice were administered an intraperitoneal glucose solution at a dose of 2 g/kg body weight [33, 34]. Blood glucose levels from the tail vein were checked at 30, 60, and 120 min after the injection through a glucometer (Yuyue, China). The AUC of OGTT was computed to assess glucose tolerance.

At E18.5, ITT was performed to evaluate insulin sensitivity. After a 6‐h fast, mice were administered insulin (0.5 U/kg, i.p.). Blood glucose was recorded at 0, 15, 30, 60, 90, and 120‐min intervals. The AUC for ITT was calculated to quantify insulin response. Prior to the ITT fasting period, fecal samples were collected from each group of mice in preparation for the subsequent FMT experiment. After the ITT, eight mice per group were euthanized to collect blood and intestinal tissue. The remaining six female mice in each group were allowed to give birth. For exploratory purposes, offspring body weight was measured at birth and after weaning (4 weeks after birth) to preliminarily evaluate intergenerational effects on growth and metabolic‐related development induced by gestational sucralose exposure.

### 2.5. Generation of Germ‐Free Mice and FMT Protocol

Female C57BL/6 J mice, aged 9 weeks, were acquired from the Zhejiang Provincial Laboratory Animal Centre and maintained under standardized housing conditions. Following a 1‐week acclimatization period, the mice received a cocktail of antibiotics (1 g/L each of ampicillin, neomycin, chloramphenicol, and metronidazole) in their drinking water for 2 weeks to deplete gut microbiota. Fecal samples were collected before antibiotic treatment and 2 weeks posttreatment cessation, and DNA quantification was performed to confirm successful microbiota depletion. For FMT, fecal samples from donor mice (CON, SUC 0.24, and SUC 0.72 groups) were homogenized in phosphate‐buffered saline by vortexing for 1 min, followed by centrifugation (500 × g, 3 min, room temperature) to obtain the supernatant.

Germ‐free mice were randomly assigned into three experimental groups (FMT‐CON, FMT‐SUC 0.24, FMT‐SUC 0.72) based on the fecal supernatants administered to them. Each mouse was administered 200 *μ*L of its assigned fecal supernatant via oral gavage daily for five consecutive days prior to mating. After conception, FMT was repeated weekly until euthanasia. OGTT and ITT were performed at embryonic days E12.5 and E18.5, respectively. Prior to the ITT fasting period, fecal samples were collected from each FMT group of mice. All mice were euthanized at E18.5 for tissue collection.

### 2.6. RNA/DNA Extraction and qRT‐PCR

Intestinal tissue RNA was extracted with a Total RNA Kit (AG, 11728) following the manufacturer′s instructions. The procedure involved reverse transcription at 37°C, continuing for 15 min, and enzyme inactivation was done at 85°C, continuing for 5 s. qRT‐PCR was carried out using SYBR RT‐PCR kits (AG, 11701). qRT‐PCR was performed to detect the expression levels of mRNA for glucose transporters (GLUT‐1, GLUT‐4) and the inflammatory factor IL‐18, which were quantified via qRT‐PCR, with normalization to *β*‐actin. The primer sequences used were as follows:

• *β*‐Actin: F:5 ^′^‐ATATCGCTGCGCTGGTCGTC‐3 ^′^, R:5 ^′^‐AGGATGGCGTGAGGGAGAGC‐3 ^′^.
• IL‐18: F:5 ^′^‐GCGTCAACTTCAAGGAAATGATGT‐3 ^′^, R:5 ^′^‐TGTCAACGAAGAGAACTTGGTCAT‐3 ^′^.
• Glut‐1: F:5 ^′^‐CAAGTTCGGCTATAACACTGGTG‐3 ^′^, R:5 ^′^‐GCCCCCGACAGAGAAGATG‐3 ^′^.
• Glut‐4: F:5 ^′^‐GTGACTGGAACACTGGTCCTA‐3 ^′^, R:5 ^′^‐CCAGCCACGGTTGCATTGTAG‐3 ^′^.

Note: Corrected primer sequences for consistency (e.g., “CAOG” → “CAAGT” in Glut‐1 forward primer).

The extraction of fecal DNA was carried out with the TIANGEN Bacterial Genomic DNA Extraction Kit (DP302), adhering closely to the instructions provided by the manufacturer.

### 2.7. Quantification of Neurotransmitters by ELISA

Blood samples were centrifuged at 4000 rpm for 15 min within 2 h of collection, and the supernatants were stored at −80°C until analysis. An ELISA kit from Meimian Industrial Co. Ltd. (MM‐0579 M2) was used to determine serum insulin concentrations, following the instructions provided by the manufacturer. The HOMA‐IR was calculated by the formula: (FBG × fasting insulin)/22.5.

### 2.8. WB Analysis

Intestinal tissue proteins were extracted and quantified. Western blot analysis was performed to assess the protein expression of tight junction markers ZO‐1 and Claudin‐1, indicative of intestinal barrier function. *β*‐Actin served as the internal reference. Intestinal tissue samples were treated with lysis buffer containing protease and phosphatase inhibitors to preserve protein integrity. Protein levels were measured using a bicinchoninic acid assay, and equivalent protein quantities were denatured in SDS loading buffer at 95°C for 10 min. Proteins were isolated using SDS‐PAGE and then transferred to a PVDF membrane through wet transfer. Using 5% BSA, nonspecific binding was inhibited, and samples were incubated with primary antibodies at 4°C for the night. Membranes were treated with HRP‐linked secondary antibodies following a wash with TBST. Protein bands were detected with an ECL kit and analyzed using ImageJ software. Target protein expression levels were standardized using *β*‐actin as a reference. Primary antibodies included anti‐Claudin‐1 (No. 28674‐1‐AP, 1:4000), anti‐ZO‐1 (No. 21773‐1‐AP, 1:5000), and anti‐*β*‐actin (No. 66009‐1‐Ig, 1:10000) (Proteintech).

### 2.9. Histological Analysis by HE Staining

Histological analysis was performed on fixed and sectioned intestinal tissues for microscopic examination. Histological changes including crypt depth and goblet cell density were analyzed to assess intestinal structural integrity. Following a 24‐h preservation in 4% paraformaldehyde, the intestinal tissue samples were embedded in paraffin. Deparaffinization of sections was done in xylene, followed by rehydration through graded ethanol and rinsing in PBS. The sections were processed by staining with hematoxylin and eosin, dehydrating with graded ethanol, clearing in xylene, and mounting with neutral resin. The histological assessment was carried out using a light microscope, and quantitative assessments of goblet cell density and intestinal crypt depth were conducted using Visiopharm image analysis software.

### 2.10. 16S rRNA Gene Sequencing and Data Analysis

Fecal DNA was extracted using the MagPure Soil DNA LQ Kit (Magan) according to the manufacturer′s instructions. DNA concentration and purity were assessed by spectrophotometry, and samples were stored at −20°C. The V3–V4 region of the 16S rRNA gene was PCR‐amplified using barcoded universal primers and high‐fidelity Takara Ex Taq DNA polymerase. Libraries were prepared with the Illumina TruSeq Nano DNA LT Library Prep Kit and validated using the Agilent High Sensitivity DNA Kit. OE Biotech Co., Ltd. (Shanghai, China) conducted the sequencing of qualified libraries, including library preparation, sequencing, and bioinformatic analysis.

Detailed methods were presented in the Supporting Information.

### 2.11. Untargeted Metabolomics Analysis and Data Processing

OE Biotech Co. Ltd. (Shanghai, China) conducted the untargeted metabolomics profiling. Fecal samples were homogenized in a 20% acetonitrile‐methanol solution with internal standards, then vortexed and centrifuged (12,000 × g, 10 min, 4°C). A 200 *μ*L sample of the supernatant was collected and stored at –20°C for 30 min. The clarified supernatant, obtained after a second identical centrifugation, was analyzed for metabolomics using UPLC‐MS/MS.

### 2.12. Statistical Analysis

Statistical analysis was executed using Version 9.4.1 of GraphPad Prism. Data are presented as mean ± SEM. Group comparisons were evaluated using methods like Student′s *t*‐test or one‐way ANOVA. The relationship between fecal 16S rRNA sequencing data and metabolomic profiles was assessed by Spearman′s correlation analysis. A *p* value under 0.05 indicated statistical significance.

## 3. Results

### 3.1. Sucralose Exposure Elevates GDM Risk

**Figure 1:**
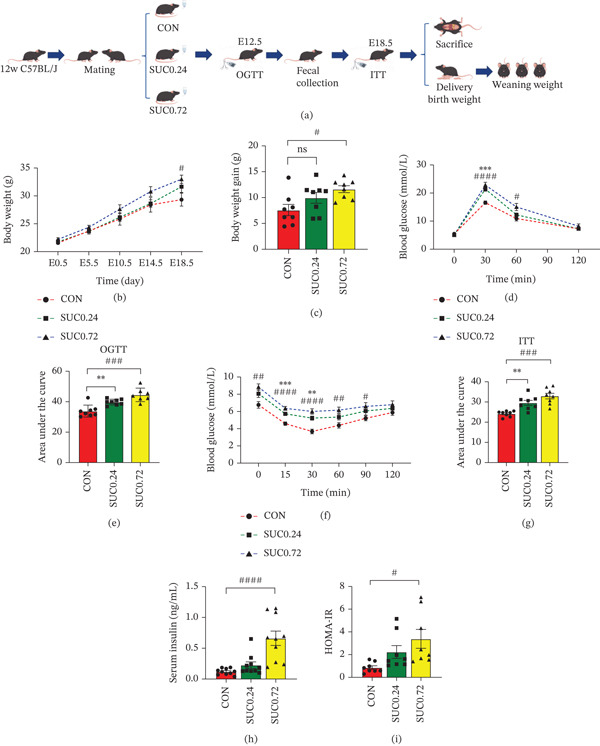
Sucralose exposure increases the risk of GDM. (a) Experimental procedure. (b) The body weight of three groups mice during pregnancy. (c) The comparison of gaining body weight of three groups mice during pregnancy. (d) The result of OGTT of three groups mice during pregnancy. (e) The result of AUC of OGTT of three groups mice. (f) The result of ITT of three groups mice during pregnancy. (g) The result of AUC of ITT of three groups mice. (h) Serum insulin levels of three groups mice. (i) HOMA‐HR levels of three groups mice. Data were shown as the mean ± SEM. Significance levels for the SUC 0.72 group versus the control group were denoted as ns for *p* ≥ 0.05, ∗ for *p* < 0.05, ∗∗ for *p* < 0.01, ∗∗∗ for *p* < 0.001, and ∗∗∗∗ for *p* < 0.0001. Significance levels for the SUC 0.72 group versus the control group were denoted as ns for *p* ≥ 0.05, # for *p* < 0.05, ## for *p* < 0.01, ### for *p* < 0.001, and #### for *p* < 0.0001. *n* = 8 per group.

The protocol for the experiment was shown in Figure 1a. Longitudinal monitoring of gestational weight revealed that mice in the SUC 0.72 group exhibited significantly greater body weight gain compared with the CON group (Figure 1b,c; *p* < 0.05), whereas the SUC 0.24 and CON groups showed no significant difference (Figure 1b,c; *p* > 0.05). OGTT analysis demonstrated elevated peak blood glucose concentrations in both sucralose‐treated groups relative to controls (Figure 1d; SUC 0.24 vs. CON at 30 min: *p* < 0.05; SUC 0.72 vs. CON at 30 and 60 min: *p* < 0.05), with corresponding increases in glucose AUC values (Figure 1e; *p* < 0.05). ITT results indicated impaired insulin sensitivity, with the SUC 0.24 group showing attenuated glucose clearance at 15 and 30 min postinjection (Figure 1f; *p* < 0.05), and the SUC 0.72 group maintaining elevated glycemia throughout the 90‐min observation period (Figure 1f; all time points *p* < 0.05). Both treatment groups displayed significant alterations in ITT AUC compared with controls (Figure 1g; *p* < 0.05). Biochemical analyses indicated hyperinsulinemia and elevated HOMA‐IR indices in the SUC 0.72 group (Figure 1h,i; *p* < 0.05), whereas the SUC 0.24 group showed no significant changes (Figure 1h,i; *p* > 0.05).

### 3.2. Sucralose Exposure Impairs Intestinal Barrier Integrity and Function in GDM

**Figure 2:**
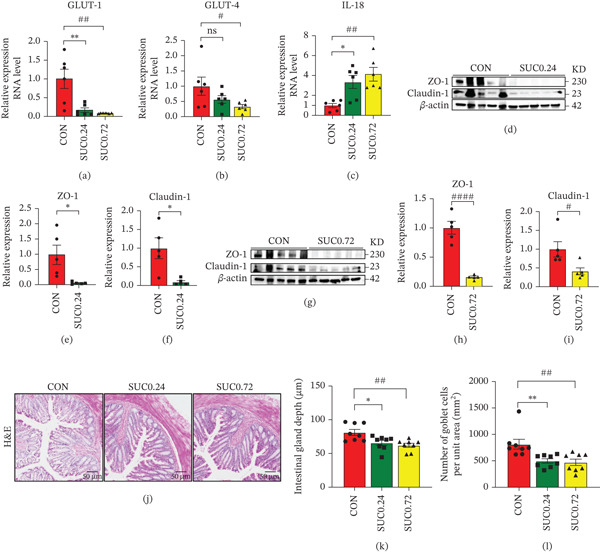
Sucrose exposure alters intestinal tight junction protein expression, glucose transporter levels, and intestinal structure in GDM. (a) The relative expression of GLUT‐1 in RNA level. (b) The relative expression of GLUT‐4 in RNA level. (c) The relative expression of IL‐18 in RNA level. (d) The expression of Claudin‐1 and ZO‐1 in protein level between CON and SUC 0.24 group. (e) Quantitative result of ZO‐1in protein level. (f) Quantitative result of Claudin‐1 in protein level. (g) The expression of Claudin‐1 and ZO‐1 in protein level between CON and SUC 0.72 group. (h) Quantitative result of ZO‐1 in protein level. (i) Quantitative result of Claudin‐1 in protein level. (j) Results of HE staining on intestinal structure (20X). (k) Quantitative result of intestinal gland depth. (l) Quantitative result of the number of goblet cells. Data were shown as the mean ± SEM. Significance levels for the SUC 0.72 group versus the control group were denoted as ns for *p* ≥ 0.05, ∗ for *p* < 0.05, ∗∗ for *p* < 0.01, ∗∗∗ for *p* < 0.001, and ∗∗∗∗ for *p* < 0.0001. Significance levels for the SUC 0.72 group versus the control group were denoted as ns for *p* ≥ 0.05, # for *p* < 0.05, ## for *p* < 0.01, ### for *p* < 0.001, and #### for *p* < 0.0001. *n* ≥ 5 per group.

Subsequently, we assessed biomarkers related to glucose metabolism and intestinal barrier integrity. Our findings indicate that sucralose exposure significantly modified the transcriptional activity of glucose transporters GLUT‐1 and GLUT‐4 and inflammatory markers IL‐18 compared with the control group (Figure 2a–c; *p* < 0.05), with a majority showing dose‐dependent responses. Protein expression of tight junction markers ZO‐1 and Claudin‐1 were notably decreased in sucralose‐treated groups compared with controls (Figure 2d–i; *p* < 0.05). Histological analysis further revealed that sucralose administration compromised intestinal architecture, as evidenced by decreased glandular depth and reduced goblet cell density (Figure 2j–l; *p* < 0.05).

### 3.3. Sucralose Exposure Alters the Gut Microbiota Composition in GDM

Fecal samples from pregnant mice were analyzed using 16S rRNA gene sequencing to assess changes in gut microbiota composition due to sucralose exposure. Alpha diversity analysis using the Shannon index revealed no significant differences in diversity between the sucralose‐exposed group and the control groups (Figure 3a; *p* > 0.05). According to the Chao1 index, high‐dose sucralose (SUC 0.72) significantly enhanced microbial diversity compared with the control group, whereas low‐dose sucralose (SUC 0.24) resulted in decreased diversity (Figure 3b; *p* < 0.05). PCoA using binary‐Jaccard dissimilarity revealed clear clustering differences in microbial communities between the control and sucralose‐treated groups, with PC1 and PC2 accounting for 11.61% and 10.55% of the variance, respectively (Figure 3c; *p* = 0.001). At the phylum level, sucralose treatment altered microbial composition: the SUC 0.24 group displayed increased Bacteroidetes and decreased Firmicutes abundance, whereas the SUC 0.72 group exhibited elevated Firmicutes levels (Figure 3d). Hierarchical clustering analysis further revealed differential enrichment of genera, including *Lactobacillus* and *Bifidobacterium*, with distinct taxonomic profiles observed in sucralose‐treated groups compared with CON (Figure 3e). In comparison to the CON group, the relative abundances of beneficial taxa like *Lachnospiraceae_UCG-001* and *Prevotellaceae_UCG-001* were significantly decreased in a sucralose dose‐dependent manner with sucralose, whereas *Parasutterella* was significantly increased in both SUC‐treated groups, with the highest increase observed in the SUC 0.24 group (Figure 3f–h). Functional prediction analysis indicated that SUC 0.24 downregulated microbial metabolic pathways, encompassing carbon metabolism, the biosynthesis of amino acids, and the creation of secondary metabolites, whereas SUC 0.72 suppressed pathways related to flavonoid degradation, thermogenesis, and N‐glycan biosynthesis (Figures 3i). LDA identified significantly enriched taxa in each group, with the top three discriminative features being o_Campylobacteraceae, f_Helicobacteraceae, and p_Campylobacter in the control group; f_Proteobacteria, f_Sutterellaceae, and *g_Parasutterella* in SUC 0.24; c_Bacilli, f_Erysipelotrichaceae, and o_Erysipelotrichales in SUC 0.72 (Figure 3j).

Figure 3Sucralose exposure remodels the gut microbiota of GDM. (a) Shannon alpha diversity (species richness) across groups. (b) Chao1 alpha diversity (species richness) across groups. (c) PCoA of binary‐Jaccard dissimilarity. (d) Relative abundance of dominant bacterial phyla. (e) Heatmap of genus‐level microbial abundance, with hierarchical clustering of taxa and groups. (f) The abundance of *Lachnospiraceae UCG-001*. (g) The abundance of *Prevotellaceae UCG-001*. (h) The abundance of *Parasutterella*. (i) PICRUSt‐predicted relative abundance of microbial biosynthetic pathways across groups. (j) LEfSe analysis: LDA scores (log10) of taxa differentially enriched in each group. *n* = 8 per group.

**Figure:**
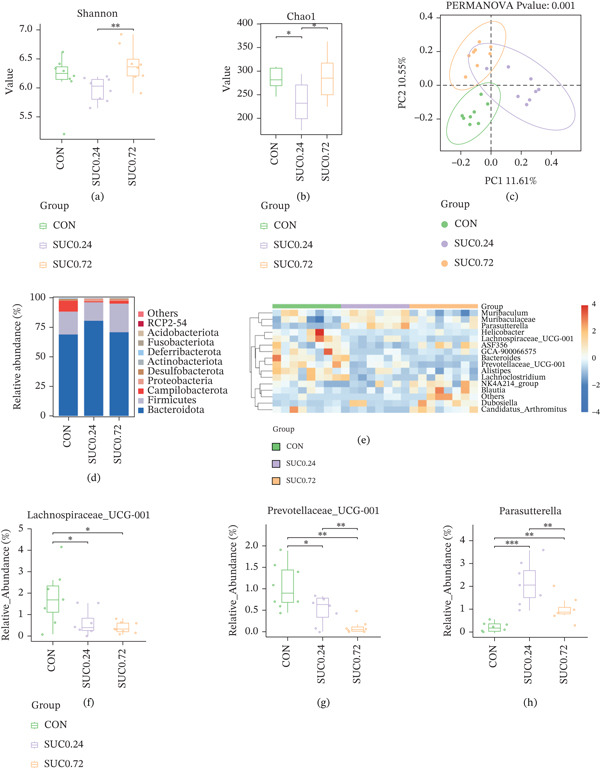

**Figure:**
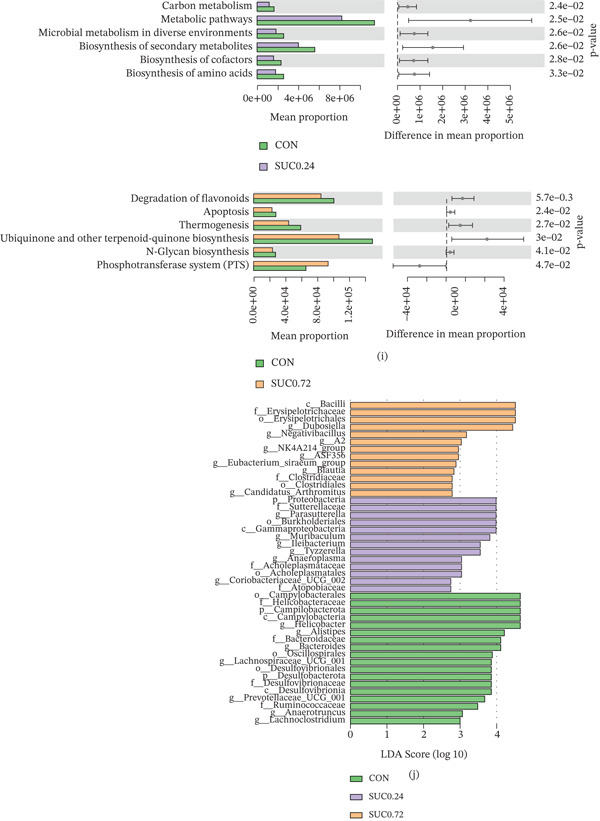

### 3.4. Sucralose Exposure Induces Alterations in Fecal Untargeted Metabolites in GDM

We performed untargeted metabolomic analysis on fecal samples from pregnant mice to assess the gut microbiota′s impact on metabolic profiles. Comparative analysis revealed 690 differentially expressed metabolites between the SUC 0.24 and CON groups, whereas 497 metabolites showed differential expression between the SUC 0.72 and CON groups. Venn diagram analysis identified 179 common differentially expressed metabolites across both comparisons (Figure 4a). PLS‐DA revealed clear group separation, with PC1 and PC2 accounting for 9.86% and 6.99% of the variance, respectively (Figure 4b). Hierarchical clustering analysis of normalized metabolite expression levels revealed distinct patterns among experimental groups (CON, SUC 0.24, SUC 0.72), with coordinated upregulation in sucralose‐exposed groups (right and middle clusters) contrasting with the control group (left cluster) (Figure 4c). Untargeted metabolomics detected three distinct sucralose intensity categories in fecal samples, showing significantly elevated levels in both SUC 0.24 and SUC 0.72 groups compared with controls (Figure 4d). KEGG pathway enrichment analysis revealed distinct metabolic disruptions, with the “Amino acid metabolism” and “Signal transduction” pathways predominantly enriched in the SUC 0.24 versus CON comparison, and the “Lipid metabolism” and “Transport and catabolism” pathways were prominent in the SUC 0.72 versus CON comparison (Figure 4e). Correlation analysis of the Top 20 differentially expressed metabolites revealed significant associations, with sucralose demonstrating strong positive or negative correlations with other metabolites (Figure 4f), suggesting its central role in metabolic regulation. Microbial‐metabolite correlation analysis identified 5‐aminopentanoic acid, L‐alloisoleucine, and LysoPC (0:0/16:0) as potential microbially regulated metabolites (Figure 4g). These findings suggest that sucralose exposure may modulate gut microbiota composition and function, potentially increasing GDM risk through subsequent metabolic alterations. Based on these findings, we propose that sucralose intake could alter gut microbiota composition, possibly increasing the risk of GDM through subsequent modifications in microbial metabolic profiles.

Figure 4Untargeted analysis of fecal metabolomics reveals distinct metabolic changes after sucralose exposure. (a) Venn diagram illustrating the overlap of differentially expressed features among three groups. (b) PLS‐DA. (c) Heatmap visualizing the expression patterns of differentially regulated features across all samples. (d) Sucralose intensity in fecal samples of these groups. (e) KEGG pathway enrichment analyses for four comparisons. (f) Correlation analysis of the Top 20 smallest *p* values differential metabolites across the three groups. (g) Correlation analysis of the Top 20 most abundant differential metabolites and microbiome across the three groups. *n* = 8 per group.

**Figure:**
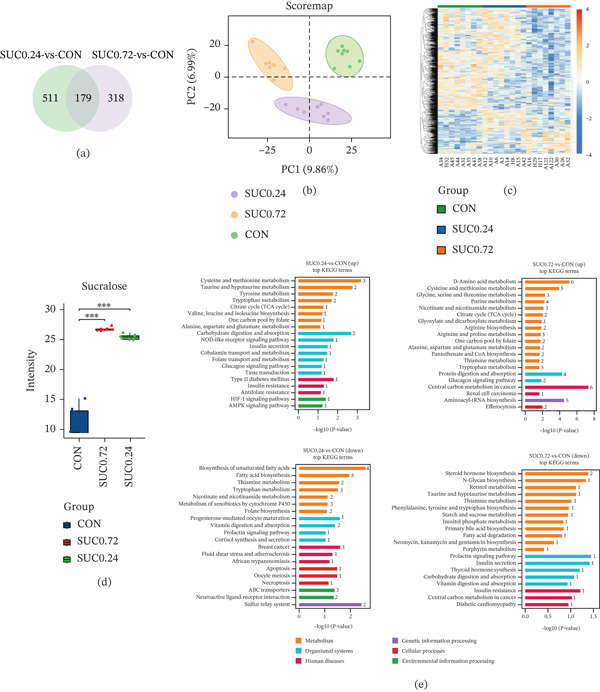

**Figure:**
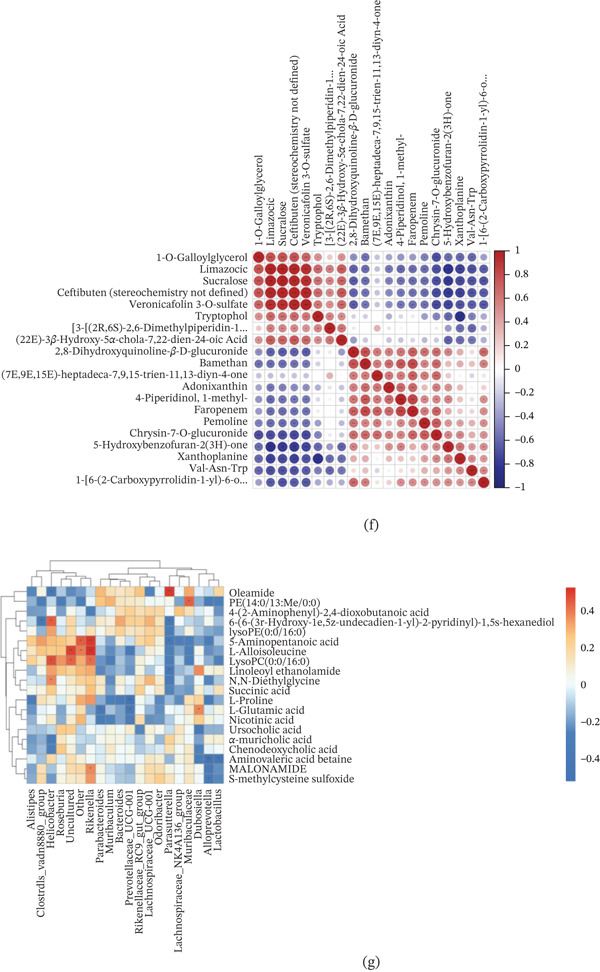

### 3.5. Mice Receiving Sucralose Fecal Microbiota Transplantation Exhibits Comparable Metabolic Phenotypes

The protocol for the experiment was outlined in Figure 5a. To clarify the role of gut microbiota in this process, FMT was conducted using samples obtained from three groups of pregnant mice. Prior to FMT, we quantified fecal DNA levels before and after establishing germ‐free mice, confirming successful colonization with a > 90% DNA clearance rate (Figure 5b). Subsequent OGTT conducted during mid‐gestation revealed that, compared with the FMT‐CON group, the FMT‐SUC 0.24 group showed a significant increase in blood glucose levels 30 min after glucose administration. Similarly, the FMT‐SUC 0.72 group displayed pronounced hyperglycemia at both 30 and 120 min (Figure 5c). Correspondingly, the AUC for OGTT was significantly increased in both FMT‐SUC groups (Figure 5d). ITT demonstrated no notable differences in glycemia between the FMT‐SUC 0.24 and FMT‐CON groups at any time point. However, the FMT‐SUC 0.72 group showed significantly higher blood glucose levels at 15, 30, and 60 min (Figure 5e). The ITT AUC analysis corroborated these findings, revealing no significant difference between FMT‐SUC 0.24 and FMT‐CON groups, but a significant elevation in the FMT‐SUC 0.72 group (Figure 5f). These metabolic profiles in FMT recipients mirrored those observed in sucralose‐exposed mice, supporting the hypothesis that sucralose modulates gut microbiota to promote GDM susceptibility.

Figure 5FMT induces phenotypic and gut microbiota alterations in recipient Mice. (a) Experimental procedure. (b) The concentrations of DNA in feces before and after germ‐free mouse establishment. (c) The result of OGTT of three FMT groups mice. (d) The result of AUC of OGTT of three FMT groups mice. (e) The result of ITT of three FMT groups mice. (f) The result of AUC of ITT of three FMT groups mice. (g) Shannon alpha diversity across FMT groups. (h) Chao1 alpha diversity across FMT groups. (i) PCoA of binary‐Jaccard dissimilarity. (j) Relative abundance of dominant bacterial phyla. (k) Heatmap of relative abundance for key gut bacterial genera/species across FMT groups. (l) LEfSe plot showing taxa with differential abundance across FMT groups. *n* ≥ 6 per group.

**Figure:**
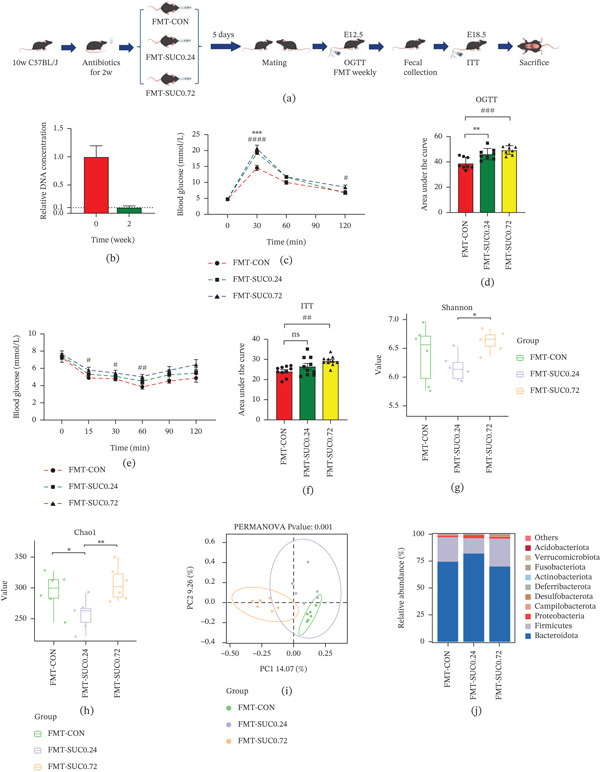

**Figure:**
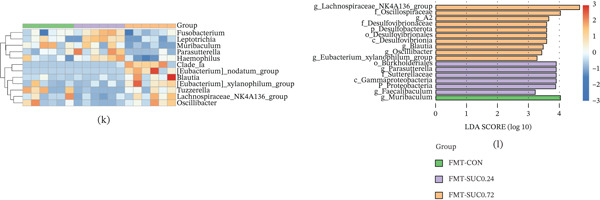

The microbial communities in FMT group were analyzed using 16S rRNA gene sequencing. Alpha diversity analysis using the Shannon index revealed no statistically significant differences in diversity between the sucralose‐exposed FMT group and the FMT‐CON group (Figure 5g; *p* > 0.05). When assessed using the Chao1 index, the FMT‐SUC 0.72 resulted in a notable increase in microbial diversity compared with the FMT‐CON group, whereas the FMT‐SUC 0.24 experienced a reduction in diversity, which was similar to the direct sucralose‐exposure mice (Figure 5h; *p*<0.05). PCoA based on binary‐Jaccard dissimilarity demonstrated distinct clustering by treatment group (*p* < 0.05), with primary separation along PC1 (14.07% variance) and PC2 (9.26% variance) (Figure 5i). Taxonomic profiling identified Bacteroidota as the dominant phylum across all groups, with subtle intergroup variations in Firmicutes and Proteobacteria abundance (Figure 5j), whose patterns were similar to those seen in mice directly exposed to sucralose. A heatmap further illustrated group‐specific variations in the standardized relative presence of specific bacterial taxa (Figure 5k). The LEfSe analysis was utilized to identify bacterial genera exhibiting the most substantial differential abundance among the FMT groups: *g_Muribaculum* in the FMT‐CON group, o_Burkholderiales in the FMT‐SUC 0.24 group, and *g_Lachnospiraceae-NK4A136-group* in the FMT‐SUC 0.72 group (Figure 5l). These findings demonstrate that FMT from sucralose‐exposed donor mice can induce GDM and alter gut microbial composition in recipient mice during pregnancy.

### 3.6. Impact of Sucralose Exposure on Maternal Postpartum Glucose Metabolism and Offspring Development in Mice

**Figure 6:**
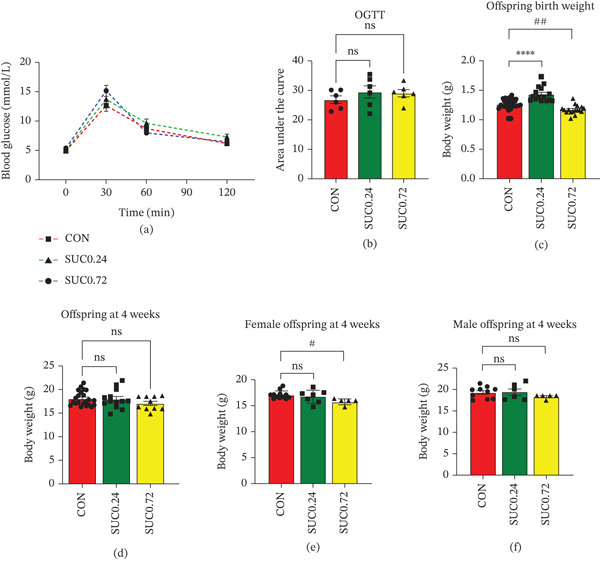
Consequence of sucralose exposure during gestation on maternal glucose metabolism after delivery and offspring′s body mass. (a) The result of postpartum 4 weeks OGTT of three groups mice. (b) The result of AUC of postpartum 4 weeks OGTT of three groups mice. (c) The body weight of offspring at birth of three groups mice. (d) The body weight of offspring at 4 weeks of three groups mice. (e) The body weight of female offspring at 4 weeks of three groups mice. (f) The body weight of male offspring at 4 weeks of three groups mice. Data were shown as the mean ± SEM. Significance levels for the SUC 0.72 group versus the control group were denoted as ns for *p* ≥ 0.05, ∗ for *p* < 0.05, ∗∗ for *p* < 0.01, ∗∗∗ for *p* < 0.001, and ∗∗∗∗ for *p* < 0.0001. Significance levels for the SUC 0.72 group versus the control group were denoted as ns for *p* ≥ 0.05, # for *p* < 0.05, ## for *p* < 0.01, ### for *p* < 0.001, and #### for *p* < 0.0001. *n* ≥ 6 per maternal group. *n* ≥ 5 per offspring group.

A thorough study assessed the impact of maternal sucralose exposure during pregnancy on postpartum glucose regulation and offspring development. Postpartum OGTT results showed no significant differences in blood glucose levels among the three groups at any time point (Figure 6a). Quantitative analysis of the AUC further confirmed the absence of statistically significant intergroup variations, consistent with the OGTT findings (Figure 6b). We observed the body weights of three offspring categories: the control group, which had 10 male and 12 female offspring; the SUC 0.24 group, which included six male and six female offspring; and the SUC 0.72 group, which consisted of five male and five female offspring. Neonatal body weight analysis demonstrated that offspring from dams exposed to low‐dose sucralose (SUC 0.24) exhibited increased birth weight compared with the CON group, whereas high‐dose exposure (SUC 0.72) resulted in reduced neonatal weight relative to controls (Figure 6c). Postweaning body weight measurements at 4 weeks revealed no notable differences between the control and either sucralose‐exposed group (Figure 6d). Yet, gender‐specific analysis showed that female offspring in the SUC 0.72 group displayed significantly lower body weight compared with CON females; such a difference was absent in males. Notably, offspring of both sexes in the SUC 0.24 group showed comparable body weights to controls (Figure 6e,f). These results indicate that although maternal sucralose consumption did not significantly alter postpartum glucose metabolism, it exerted sex‐specific effects on offspring growth parameters, with high‐dose exposure particularly influencing female offspring development.

## 4. Discussion

This study explored the association between maternal sucralose consumption and the likelihood of GDM in a murine model. Through comprehensive analyses, including gestational OGTT, ITT, intestinal histomorphological and barrier function assessments, gut microbiome genomic sequencing, metabolomic profiling, and FMT studies, we demonstrated that prenatal sucralose exposure induces maternal GDM via a cascade of microbial‐mediated mechanisms. These mechanisms include gut microbiota imbalance, compromised intestinal barrier, and resulting metabolic disturbances. Furthermore, such exposure was found to affect transgenerational development, with a pronounced effect observed in female offspring. This research, to our awareness, is the first to provide mechanistic evidence establishing a causal relationship between sucralose intake, gut microbial alterations, and GDM pathogenesis, thereby offering novel insights into the interplay between dietary additives, microbiome dynamics, and metabolic disturbances during pregnancy.

As far as we know, this study represents the first demonstration that gestational sucralose exposure increases the risk of GDM in murine models. Our findings reveal that both low‐ and high‐dose sucralose consumption during pregnancy induces glucose metabolism dysregulation and promotes mid‐gestational insulin resistance, potentiating GDM development. These results challenge the conventional view of artificial sweeteners as metabolically inert and align with emerging clinical evidence. A Chilean cohort study (*n* = 1472) reported significant associations between sucralose intake and elevated GDM risk [13], whereas a hospital‐based investigation in Guangdong (*n* = 422) documented a 56.90% GDM incidence among high artificial sweetener consumers, demonstrating a robust dose‐response relationship (OR = 2.66, 95% CI 1.48–1.78) [19]. Although prior studies have implicated sucralose in gut microbiota dysbiosis and glucose homeostasis impairment in murine models [35, 36], its gestational effects remain understudied. GDM, the most prevalent pregnancy‐related metabolic disorder, exhibits strong correlations with gut microbial imbalance. Current evidence indicates that GDM patients display reduced gut microbial *α*‐diversity and characteristic phylum‐level alterations, including increased Firmicutes abundance with concomitant decreases in Bacteroidetes, and Actinobacteria [37, 38]. Clinical studies further suggest that dietary modifications may improve glycemic control in GDM through microbiota‐mediated mechanisms. Notably, our high‐dose sucralose‐exposed GDM mice recapitulated these microbial shifts, exhibiting increased Firmicutes and decreased Bacteroidetes abundance. These findings collectively demonstrate that gestational sucralose exposure induces gut dysbiosis resembling that observed in GDM patients. Coupled with clinical evidence linking sucralose consumption to GDM risk, our results position artificial sweeteners as potential environmental contributors to GDM pathogenesis, offering novel insights for prenatal nutritional guidance.

The study demonstrates that sucralose‐induced gut microbiota remodeling constitutes a pivotal mechanism in GDM progression. This conclusion is substantiated by converging evidence from both preclinical and clinical investigations. Specifically, chronic sucralose consumption at FDA‐ADI levels has been shown to significantly reduce the abundance of beneficial bacterial taxa, including *Bifidobacterium* and *Lactobacillus* [32]. Furthermore, sucralose not only promotes the proliferation of proinflammatory microbial populations but also modulates host immune responses through microbial metabolite alterations [39, 40]. Compelling evidence indicates that sucralose disrupts intestinal barrier integrity and exacerbates low‐grade intestinal inflammation by altering key gut microbial metabolites [25, 41], which is consistent with our results. FMT experiments established a causal relationship between gut microbiota dysbiosis and the development of GDM. Notably, FMT from sucralose‐exposed mice successfully recapitulated the GDM phenotype, corroborating findings from gut that demonstrated glucose metabolism abnormalities in germ‐free mice following microbiota transfer from GDM patients [42]. Mechanistically, sucralose exposure downregulated Claudin‐1 and ZO‐1, increased intestinal permeability, and reduced expression of glucose transporters (GLUT1 and GLUT4), concomitant with elevated IL‐18 levels. These observations align with prior studies implicating NF‐*κ*B‐mediated inflammatory pathways in insulin [43–45]. Fecal metabolomic analysis confirmed substantial sucralose accumulation in feces, indicating minimal systemic absorption [46]. Correlation analysis of the Top 20 metabolites revealed significant associations between sucralose and multiple microbial‐derived metabolites, suggesting that despite its nonabsorbable nature, sucralose undergoes microbial metabolism, contributing to metabolic dysregulation. These results indicate that sucralose‐induced dysbiosis could harm the function of the intestinal barrier and disrupt metabolic homeostasis, potentially contributing to GDM development through a dual‐pathogenic mechanism.

Beyond investigating the role of sucralose in GDM, this study also examined its influence on the body weight of GDM offspring. Our findings revealed a dose‐dependent effect of prenatal sucralose exposure on offspring birth weight. Specifically, low‐dose exposure increased birth weight, whereas high‐dose exposure resulted in a reduction. Over time, the effect of sucralose on offspring body weight attenuated progressively. Four weeks after weaning, offspring mice groups showed no significant differences in body weight. However, female offspring exposed to high‐dose sucralose exhibited persistently lower body weight compared with controls, suggesting potential sex‐specific responses to sucralose exposure. Currently, no studies have specifically addressed the effects of prenatal sucralose exposure on GDM offspring. Existing research on artificial sweetener consumption during pregnancy is limited. A Canadian cohort study (*n* = 3033 mother‐infant pairs) demonstrated that daily maternal intake of artificial sweeteners during pregnancy doubled the risk of infant overweight by 1 year of age [47]. Similarly, a Danish study (*n* = 918 mother‐child pairs) found that daily artificial sweetener consumption during pregnancy was associated with increased gestational age at birth and a higher likelihood of childhood overweight or obesity by age seven [48]. These findings indirectly support the hypothesis that maternal metabolic perturbations induced by artificial sweeteners may influence offspring phenotypes. The present study provides valuable insights for future clinical monitoring of long‐term metabolic consequences in children born to mothers who consumed sucralose during pregnancy.

This study exhibits several notable strengths. Firstly, the investigation specifically targeted the physiological phase of pregnancy, with a focused examination of sucralose rather than a broad analysis of artificial sweeteners. To the best of our understanding, this represents the first systematic confirmation of the association between sucralose exposure and GDM risk through preclinical research, thereby addressing a critical gap in foundational studies on artificial sweetener consumption during pregnancy. Secondly, the study elucidated a multidimensional cascade mechanism, providing the first systematic validation of a causal relationship between sucralose and GDM risk. Thirdly, FMT experiments yielded compelling evidence supporting the pivotal mediating role of gut microbiota, offering a comprehensive and methodologically rigorous demonstration of this causal link. Additionally, by focusing on GDM—a clinically prevalent pregnancy complication—the findings furnish direct scientific evidence to guide dietary recommendations during pregnancy, including potential warnings regarding artificial sweetener risks, with clear translational implications for clinical practice.

However, this study is not without limitations. Firstly, the exclusive reliance on murine models, without incorporation of clinical population data, may constrain the generalizability of the conclusions to human dietary guidelines. Secondly, although the study established associations between metabolites and microbiota, it did not confirm direct causality or explore the molecular mechanisms underlying intestinal barrier dysfunction. Future investigations should aimed at elucidating these fundamental mechanisms to enhance the mechanistic depth of the findings. Thirdly, owing to the limited number of cages per group (*n* = 2), only mean values of water intake and daily sucralose intake were calculated and presented; statistical analysis was not performed in the present study, which will be addressed in future follow‐up studies. Furthermore, fecal samples were collected only in the third trimester (E18.5). The inclusion of longitudinal sampling at earlier time points (pre‐pregnancy, mid‐gestation) in future studies would help delineate the temporal dynamics of sucralose‐induced microbial shifts and their relationship to the onset of metabolic dysfunction.

## 5. Conclusion

In summary, this study systematically demonstrated that maternal sucralose exposure during pregnancy increases the risk of GDM in mice by influencing the gut microbiota composition, disrupting the intestinal barrier function, and modulating metabolic profiles. Furthermore, it revealed transgenerational effects on offspring body weight, with a pronounced impact observed in female progeny, thereby offering novel insights into environmental determinants and intergenerational GDM prevention strategies. Future research should investigate the dose‐response relationship and clinical translatability of these findings to provide evidence‐based dietary recommendations for pregnancy and metabolic disorder prevention.

**Nomenclature**
ADI: approved acceptable daily intake
AUC: area under the curve
BSA: bovine serum albumin
EFSA: European Food Safety Authority
FBG: fasting blood glucose
FDA: Food and Drug Administration
FMT: fecal microbiota transplantation
GDM: gestational diabetes mellitus
HE: hematoxylin and eosin
HED: human equivalent dose
HOMA‐IR: homeostasis model assessment of insulin resistance
ITT: insulin tolerance test
LDA: linear discriminant analysis
LEfSe: linear discriminant analysis effect size
NNS: non‐nutritive sweeteners
OGTT: oral glucose tolerance test
PBS: phosphate‐buffered saline
PC: principal components
PCoA: principal coordinates analysis
PLS‐DA: partial least‐squares discriminant analysis
qRT‐PCR: quantitative real‐time PCR
TBST: tris‐buffered saline and tween‐20
UPLC‐MS/MS: ultra‐performance liquid chromatography‐tandem mass spectrometry
WB: western blot

## Author Contributions

**Jiajia Song**: methodology, formal analysis, visualization, software, writing—original draft. **Juhui He**: investigation, data curation, methodology, formal analysis. **Zhaoxia Liang**: conceptualization, project administration, resources, funding acquisition, writing—review and editing. Jiajia Song and Juhui He are co‐first authors.

## Funding

This study was supported by grants from National Natural Science Foundation of China (Grant Number 82571935); 4 + X Clinical Research Project of Women′s Hospital, School of Medicine, Zhejiang University (Grant Number ZDFY2022‐4XB101); and Joint TCM Science & Technology Projects of National Demonstration Zones (Grant Number GZY‐KJS‐ZJ‐2025‐037).

## Disclosure

All authors have read and approved the final version of the manuscript. Corresponding author had full access to all of the data in this study and takes complete responsibility for the integrity of the data and the accuracy of the data analysis.

## Conflicts of Interest

The authors declare no conflicts of interest.

## Supporting information


**Supporting Information** Additional supporting information can be found online in the Supporting Information section. Materials and methods for 16S rRNA. Table S1: Daily sucralose intake and human equivalent dose (HED) in mice across experimental groups.

## Data Availability

The authors confirm that the data supporting the findings of this study are available within the article and/or its supporting information.
