# Supplementary material for: Sucralose Exposure During Pregnancy Elevates Gestational Diabetes Risk via Gut Microbiota‐Metabolic Axis in Mice
Source: J Diabetes Res. 2026 May 13;2026:8638903. doi: 10.1155/jdr/8638903 (PMC13170706; doi:10.1155/jdr/8638903)
Supplement: Supplementary file 1 — Supporting Information Additional supporting information can be found online in the Supporting Information section. Materials and methods for 16S rRNA. Table S1: Daily sucralose intake and human equivalent dose (HED) in mice across experimental groups. [file JDR-2026-8638903-s001.docx]

**Supporting Information**

**Sucralose Exposure During Pregnancy Elevates Gestational Diabetes Risk via Gut Microbiota-Metabolic Axis in Mice**

Jiajia Song^#1,2^, Juhui He^#1,2^, Zhaoxia Liang^*1,2^

1. Department of Obstetrics, Women’s Hospital, School of Medicine, Zhejiang University, Hangzhou 310006, China

2. Zhejiang Key Laboratory of Maternal and Infant Health, Hangzhou, China

**^#^ Co-First Author**

**^*^Corresponding Author:**

**Zhaoxia Liang**, MD, PhD.

Department of Obstetrics, Women’s Hospital, School of Medicine, Zhejiang University, Hangzhou 310006, China; E-mail: xiaozaizai@zju.edu.cn.

Summary: 3 pages, 1 table.

**1. Materials and methods for 16S rRNA**

Fecal samples were subjected to 16S rRNA gene sequencing. Genomic DNA was extracted using the MagPure Soil DNA LQ Kit (Magan) according to the manufacturer’s instructions. DNA concentration and purity were examined by spectrophotometry, and extracts were stored at -20°C. The V3-V4 hypervariable region of the 16S rRNA gene was amplified by PCR using barcoded specific primers and high-fidelity Takara Ex Taq DNA polymerase. Sequencing libraries were constructed with the Illumina TruSeq Nano DNA LT Library Prep Kit, and library quality was validated using the Agilent High Sensitivity DNA Kit. Qualified libraries were sequenced on an Illumina platform.

All library preparation, sequencing, and bioinformatic analysis were performed by Shanghai Ouyi Biomedical Technology Co., Ltd. Raw data were delivered in FASTQ format. Primer sequences were removed using Cutadapt. High-quality reads were processed for quality filtering, denoising, assembly, and merging via the DADA2 plugin in QIIME 2 (November 2020) with default parameters to generate amplicon sequence variants (ASVs) and the corresponding ASV abundance table. Representative sequences of each ASV were taxonomically annotated against the SILVA database (version 138) using the q2-feature-classifier with default parameters.

Alpha diversity was assessed using the Chao1 and Shannon indices. Beta diversity was analyzed by principal coordinate analysis (PCoA) based on unweighted UniFrac distance matrices calculated in R. Group differences were examined using Student’s t-test, one-way ANOVA, Kruskal–Wallis test, and Wilcoxon test as appropriate. Linear discriminant analysis effect size (LEfSe) was used to identify differentially abundant taxa across groups. All procedures, including library preparation, sequencing, and bioinformatic analysis, were conducted by Ouyi Biomedical Technology Co., Ltd. (Shanghai, China).

**Table S1. Daily sucralose intake and human equivalent dose (HED) in mice across experimental groups.**

|  | **Experimental group** | | |
| --- | --- | --- | --- |
|  | **CON (n = 10)** | **SUC 0.24 (n = 7)** | **SUC 0.72 (n = 10)** |
| Mean BW in E5.5 (g) | 21.47 ± 0.28 | 21.92 ± 0.30 | 22.24 ± 0.25 |
| Mean BW in E12.5 (g) | 25.64 ± 0.87 | 27.6 ± 0.34 | 27.81 ± 0.66 |
| Mean BW (g) | 23.56 ± 0.55 | 24.76 ± 0.21 | 25.02 ± 0.32 |
| Weekly water intake (g) | 39.95 | 26.99 | 49.07 |
| Average water intake  (g/mouse/day) | 4.00 | 5.28 | 4.91 |
| Daily sucralose intake  (mg/kg/day) | 0 | 51.18 | 141.29 |
| HED (mg/kg) | 0 | 4.81 | 13.28 |

Note: Each group was housed in 2 cages, statistical comparison was not feasible. Daily sucralose intake was calculated from weekly water intake and mean body weight. Abbreviations: BW, body weight; SEM, standard error of the mean. HED, human equivalent dose. SUC: sucralose. Values were presented as mean (no SEM available due to limited cage replicates)
